# Juvenile myasthenia gravis in Norway: HLA-DRB1*04:04 is positively associated with prepubertal onset

**DOI:** 10.1371/journal.pone.0186383

**Published:** 2017-10-16

**Authors:** T. H. Popperud, M. K. Viken, E. Kerty, B. A. Lie

**Affiliations:** 1 Department of Neurology, Oslo University Hospital, Oslo, Norway; 2 Institute of Clinical Medicine, University of Oslo, Oslo, Norway; 3 Unit for hereditary and inborn neuromuscular disorders, Department of Neurology, Oslo University Hospital, Oslo, Norway; 4 Department of Immunology, University of Oslo and Oslo University Hospital, Oslo, Norway; 5 Department of Medical Genetics, University of Oslo and Oslo University Hospital, Oslo, Norway; Boston Children's Hospital / Harvard Medical School, UNITED STATES

## Abstract

**Background:**

Juvenile myasthenia gravis (MG) is a rare autoantibody mediated autoimmune disorder targeting the neuromuscular endplate. The clinical hallmark is muscle weakness and fatigability. Disease aetiology is complex, including both genetic and environmental factors. The involvement of genes in the human leukocyte antigen (HLA) is well established in adult MG. However, HLA associations in European juvenile MG have not been studied. This case-control study aimed to investigate and characterize genetic risk factors in prepubertal and postpubertal onset juvenile MG.

**Methodology/Principal findings:**

A population based Norwegian cohort of 43 juvenile MG patients (17 with prepubertal onset, 26 with postpubertal onset) and 368 controls were included. Next generation sequencing of five HLA loci (HLA-A, -B, -C, -DRB1 and -DQB1) was performed, and a positive association was seen with HLA-B*08 (OR (95% CI) = 3.27 (2.00–5.36), P_c_ = 0.00003) and HLA-DRB1*04:04 (OR (95% CI) = 2.65 (1.57–4.24), P_c_ = 0.03). Stratified in postpubertal and prepubertal onset, HLA-DRB1*04:04 was only positively associated with the latter (P = 0.01). The HLA-B*08 allele (12.9% in the controls), previously described associated with early onset adult MG, was most frequently observed in postpubertal onset MG (40.4%, P = 0.0002) but also increased among prepubertal onset MG (23.5%, P = 0.05).

**Conclusion:**

This study provides novel information about HLA susceptibility alleles in Norwegian juvenile MG where HLA-DRB1*04:04 was associated with prepubertal onset.

## Introduction

Myasthenia gravis (MG) is a rare autoimmune disorder affecting the neuromuscular endplate. The disease can occur at any age, and when onset in childhood it is termed juvenile MG. The upper age-cut off for juvenile MG varies between studies, but is often set at age ≤ 18 years [1]. Due to its heterogeneous nature, MG is in addition to age at onset, sub classified according to clinical presentation (ocular MG vs generalised MG), thymus histopathology (thymoma, thymus hyperplasia) and autoantibody (ab) profile (acetylcholine receptor (AChR) ab, muscle-specific kinase (MuSK) ab, lipoprotein receptor-related protein 4 (Lrp4) ab) [2]. In the majority of cases autoantibodies are directed towards the AChRs [3, 4]. The immune response, which is B cell mediated, T cell dependent and also involving complement factors, leads to impaired neuromuscular transmission, and the MG patients experience intermittent and fatigable weakness of skeletal muscles [5].

The disease aetiology in MG is complex and multifactorial involving both genetic and environmental factors [6]. MG does not show a strong heritability, and the frequency of familial MG cases is reported to be low, from 3–7% [7–9]. However, co-occurrence of autoimmune diseases among family members of MG patients [10, 11], the fact that clinical manifestations of MG differ between racial groups [12] and a higher concordance rate of MG among monozygotic compared to dizygotic twins[13], suggests an influence by genetic factors in disease pathogenesis. The strongest genetic determinant is the involvement of genes in the human leukocyte antigen (HLA) complex located on the short arm of chromosome 6 [14, 15]. The associated HLA alleles vary with the different MG subgroups and with racial origin. In European populations, an association with HLA-B*08 in early onset MG (EOMG, onset < 40 years) have been shown [14, 16], while the strongest HLA risk allele in late onset MG (LOMG, onset >60 years) is DRB1*15:01 allele [16, 17]. None of the European studies have focused on juvenile MG. However, in East-Asian populations where juvenile MG is more frequent, HLA genes have been studied in this subgroup. A positive association has been found with the HLA-B*46 -DRB1*09 haplotype and ocular juvenile MG in a Chinese population [18], and with the HLA-DRB1*1302 haplotype and latent generalized juvenile MG in a Japanese population [19].

Studies on clinical presentation suggest that juvenile MG, and especially when prepubertal onset, differ from adult MG in some aspects such as autoantibody status and disease severity [1, 20].

This study aimed to investigate and characterize genetic risk factors in a cohort of Norwegian juvenile MG patients through comprehensive genotyping of HLA class I and II loci, and in particular, whether there were specific HLA risk alleles in the prepubertal onset subgroup.

## Methods

### Patients

In this population-based study, juvenile MG cases were identified from Jan 2012 to Apr 2016, through multiple strategies: i) through neurological and/or paediatric departments at the 15 main hospitals in Norway, ii) through the national AChR ab database at Haukeland University Hospital and iii) through the national adult MG database at Oslo University Hospital. Inclusion criteria were acquired MG with typical clinical symptoms and onset ≤ 18 years of age, in addition to AChR antibody positivity and/or neurophysiologic findings consistent with MG (pathological decrement after repetitive nerve stimulation and/or increased jitter on single-fibre electromyogram)[21].

A total of 75 juvenile MG patients were identified, and 53 (71%) gave consent to participate and donate a blood sample. Inclusion criteria were verified through patients´ medical records and the following additional clinical information were registered: gender, age at onset, thymectomy status, thymus histology and co-occurring autoimmune disorders. A more detailed description of the clinical characteristics in the Norwegian JMG cohort is given in a previous publication [22].

To avoid possible population stratification, only ethnically Norwegian cases were included. Hence, eight non-ethnic Norwegians were excluded, as well as one MuSK positive patient and one out of a pair of siblings. No thymoma patients were found.

All together 43 unrelated ethnic Norwegian juvenile MG patients were included in the study. The patients were divided into prepubertal onset group when MG onset age < 12 years, and postpubertal onset group when MG onset age 12–18 years. In addition we registered age at menarche in all female patients, and all cases defined as prepubertal onset had MG onset before menarche.

### Controls

Previously genotyped HLA-data for 368 healthy controls, randomly selected among Norwegian bone marrow donors recruited through the Norwegian Bone Marrow Donor Registry (http://www.nordonor.org/), were utilized [23, 24]. 30% were female and 70% were male, however, no difference in the HLA allelic distribution was seen between female and male controls.

### HLA genotyping

Genomic DNA was isolated from blood samples with Gentra Autopure LS (Qiagen, Hilden, Germany). For HLA-genotyping, the NGSgo kit from GenDx (Utrecht, The Netherlands) was used to sequence the HLA-A, -B, -C, -DRB1 and -DQB1 genes with MiSeq Reagent Kit v2 (300-cycles) and 2 x 150 paired-end sequencing on an Illumina MiSeq (Illumina, San Diego, USA). The sequencing was performed at the Norwegian Sequencing Centre (NSC, www.sequencing.uio.no), University of Oslo, Norway. The sequencing results were analysed and HLA-genotypes obtained by using the NGSengine v2.1 analysis software (GenDx).

### Statistics

Statistical analysis of genetic associations was performed using UNPHASED v.3.0.10 [25]. Rare alleles (n<2 in both patients and controls) were excluded. The expectation maximization algorithm was used to estimate maximum likelihood haplotype frequencies. The haplotype method [26] and the Svejgaard method [27] were used to assess which alleles and loci showed the primary association and which appeared to be secondary due to linkage disequilibrium (LD). Values for D’ and r^2^ were calculated for allele combinations. Odds ratios (OR) and 95% confidence intervals (CI) were calculated with Woolf’s formula comprising Haldane’s correction. P_c_ values <0.05 after correction for number of comparisons in the initial global locus tests (n = 5) were considered significant. For allelic associations, we present both uncorrected P values (P_nc_) and P-values corrected (P_c_) for the number of tested alleles at each locus (n = 8 for HLA-A, n = 9 for HLA-B, n = 9 for HLA-C, n = 10 for HLA-DRB1, and n = 10 for HLA-DQB1). We did not correct for the total number of alleles tested, as the alleles at HLA loci do not fully represent independent tests due to the strong LD.

### Ethics

The study was approved by the Norwegian Regional Committee for Medical and Health Research Ethics, South East Office. All patients, or their parents when underage, gave written informed consent. Data was collected and registered in accordance with Norwegian guidelines.

## Results

The main clinical characteristics of the 43 unrelated ethnic Norwegian juvenile MG patients included in the study are listed in **Table 1**. Age at onset ranged from 1 year to 18 years, 17 had prepubertal onset and 26 postpubertal onset.

**Table 1:** Clinical characteristic of the Norwegian juvenile myasthenia gravis cohort stratified by age at onset.

	PREPUBERTAL ONSET (n = 17), n (%)	POSTPUBERTAL ONSET (n = 26), n (%)
**Female (n = 35)**	13 (76%)	22 (85%)
**GMG (n = 40)**	16 (94%)	24 (92%)
**AChR ab + (n = 31)**	9 (53%)	22(85%)*
**HP/TX (n = 20/32)**	5/9 (56%)	15/23 (65%)
**CAD (n = 12)**	5 (29%)	7 (27%)

*Compared with prepubertal onset, P<0.05.

CAD = co-occurring autoimmune disorder other than myasthenia gravis. GMG = Generalised myasthenia gravis. AChR ab = acetylcholine receptor antibodies. HP = Thymus hyperplasia. TX = thymectomy

All patients included in the study were successfully genotyped for the five HLA loci (HLA-A, B, C, DRB1 and DQB1). Genotype success rate among controls were above 95% for all HLA loci. At locus level, two of the HLA genes were significantly associated with juvenile MG; i.e. HLA-B (P_c_ = 0.004) and HLA-DRB1 (Pc = 0.00003). The frequencies of HLA alleles significantly increased or decreased among the juvenile MG patients compared to the controls are shown in **Table 2**.

**Table 2:** HLA alleles showing association (P_nc_<0.05) with juvenile myasthenia gravis (JMG).

HLA ALLELE	JMG (n = 43) n (%)	CONTROLS (n = 368) n (%)	P_nc_	OR (95% CI)	P_c_
A*01	27 (31.4%)	119 (16.2%)	0.0005	2.38 (1.46–3.88)	0.005
A*02	12 (19.8%)	240 (32.7%)	0.02	0.52 (0.30–0.89)	ns
B*08	28 (32.6%)	94 (12.9%)	0.000003	3.27 (2.00–5.36)	0.00003
B*40	17 (19.8%)	75 (10.3%)	0.007	2.18 (1.23–3.85)	ns
C*07	42 (48.8%)	246 (34.8%)	0.01	1.78 (1.14–2.79)	ns
DRB1*03:01	26 (30.2%)	106 (14.5%)	0.0002	2.56 (1.57–4.24)	0.002
DRB1*04:01	4 (4.7%)	92 (12.6%)	0.04	0.38 (0.15–0.96)	ns
DRB1*04:04	13 (15.1%)	47 (6.4%)	0.003	2.65 (1.40–5.04)	0.03
DQB1*02:01	26 (30.2%)	137 (19.3%)	0.02	1.83 (1.12–2.98)	ns
DQB1*03:01	4 (4.7%)	117 (16.5%)	0.006	0.28 (0.11–0.69)	ns

Odds Ratio (OR) and 95% confidence interval (CI) are shown for uncorrected P values (P_nc_). P-values corrected for the number of tested alleles at each locus (Pc)

A positive association was observed between juvenile MG and alleles on the well-established ancestral haplotype 8.1 (AH8.1; A*01-B*08-C*07-DRB1*03:01-HLA-DQB1*02:01). The strongest positive association was seen with HLA-B*08 (OR (95% CI) = 3.27 (2.00–5.36), P_c_ = 0.00003), and only B*08 together with A*01 and DRB1*03:01 were significant on AH8.1 after correction for multiple testing. These three alleles occurred on a susceptibility haplotype A*01-B*08-DRB1*03:01 present in 19.8% of cases compared to 8.0% of controls (P = 0.0004). Conditional haplotype analysis showed that HLA-B*08 conferred the strongest association (p<0.02) while neither HLA-DRB1*03 nor A*01 were associated in the absence of HLA-B*08 (p>0.3).

In addition, a positive association between juvenile MG and HLA-DRB1*04:04 was observed (OR (95% CI) = 2.65 (1.57–4.24), P_c_ = 0.03). Several alleles were also found to be negatively associated; however, none of these remained significant after correction for multiple testing.

When stratifying the juvenile MG patients into prepubertal onset and postpubertal onset groups, we found that only prepubertal onset juvenile MG was associated with the HLA-DRB1*04:04 allele (P = 0.01), where it occurred in 26% compared to 6.4% among controls. HLA-DRB*04:04 was observed in 7.7% among the postpubertal onset MG, not increased compared to the controls (P = 0.3) and significantly less frequent than in the prepubertal onset MG (P = 0.01). HLA-B*08 was associated with both groups, but most pronounced with the postpubertal onset juvenile MG cases (P = 0.0002) where it was present in 40.4% compared to 23.5% in the prepubertal onset cases, and 12.9% in the controls.

Next, we compared the clinical characteristics of all the juvenile MG patients carrying the two risk alleles, HLA-B*08 and HLA-DRB1*04:04. Among the HLA-B*08+ cases, AChR antibodies and thymus hyperplasia were more frequent and age of onset higher, compared to the HLA-B*08- cases (**Table 3**). When comparing the clinical characteristics in the prepubertal onset group sub classified according to risk allele association, the main difference was age at onset (**Table 4**). In the HLA-DRB1*04:04+/HLA-B*08- cases, the median age at onset was 5 years compared to 9 years among the HLA-DRB1*04:04-/HLA-B*08+ cases.

**Table 3:** Clinical characteristics of the juvenile myasthenia gravis cohort stratified by HLA-B*08 association.

		HLA-B*08+ (n = 27)	HLA-B*08- (n = 16)
**Median age at onset in years**		14	8.5
**Age at onset**	<12 years	8 (30%)	9 (56%)
	≥12 years	19	7
**AChR ab status**	Positive	23 (85%)*	8 (50%)
	Negative	4	8
**Gender**	Female	24 (89%)	11 (69%)
	Male	3	5
**Thymectomy**	Yes	24 (89%) **	8 (50%)
	No	3	8
**Thymus histology**	Yes	18 (75%) ***	2 (13%)
	No	4	4
	Unknown	2	2
**CSR**	Yes	13 (48%)	8 (50%)
	No	14	8
**CAD**		9 (33%)	3 (19%)
**GMG**		25 (93%)	15 (93%)

*Compared with HLA-B08-, p = 0.02.

**Compared with HLA-B*08, p = 0.008.

***Compared with HLA-B*08, p = 0.005.

CAD = co-occurring autoimmune disorder other than myasthenia gravis. AChR ab = acetylcholine receptor antibodies. CSR = complete stable remission. GMG = generalised myasthenia gravis

**Table 4:** Clinical characteristics of prepubertal onset myasthenia gravis stratified by HLA association.

	DRB1*04:04+/B*08-, n(%)	DRB1*04:04-/B*08+, n(%)	DRB1*04:04+/B*08+, n(%)	DRB1*04:04-/B*08-, n(%)
**Total (n = 17)**	7	7	1	2
**Median age at onset in years**	5	9	1	2
**Female**	5 (72%)	6 (86%)	1 (100%)	1 (50%)
**AChR ab**	3 (43%)	5 (72%)	1 (100%)	0
**HP/TX**	1/3	3/5	1/1	0/0
**CAD**	2 (29%)	2 (29%)	1	0
**CSR**	4 (71%)	4 (71%)	1 (100%)	1 (50%)

AChR ab = acetylcholine receptor antibodies. HP = Thymus hyperplasia. TX = Thymectomy. CAD = co-occurring autoimmune disorder other than myasthenia gravis. CSR = complete stable remission

The HLA-B*46:01 allele reported in Asian juvenile MG populations was not detected in our juvenile MG cohort, and the HLA-DRB1*09:01 was present at a low frequency both in patients and controls, 1.2% and 0.8% respectively. The DRB1*13:02 allele reported to be associated with latent generalized juvenile MG in Japan, was insignificantly (P_nc_ = 0.1) increased among our juvenile MG population (10.5%) vs controls (5.3%).

## Discussion

The association of specific HLA alleles with MG has been known for decades [9], but this is to our best knowledge the first study on HLA associations in European juvenile MG patients. We investigated HLA class I and II alleles in a nationwide Norwegian cohort of MG patients with juvenile disease onset, exploring potential risk alleles in this MG subgroup and identified a positive association with HLA-B*08 and HLA-DRB1*04:04. The HLA-B*08 association is well established in European EOMG[14], while the association with HLA-DRB1*04:04 is a new finding not earlier described in MG patients. However, in other autoimmune disorders like rheumatoid arthritis and autoimmune Addison’s disease, especially in early onset cases, an association with HLA-DRB1*04:04 has been shown[28–30].

Interestingly, we found that the HLA associations were differed according to the age at MG onset. The HLA-B*08 was most frequent in the cases with postpubertal onset, while the HLA-DRB1*04:04 was only positively associated with prepubertal onset.

In the prepubertal onset group, the main difference between the HLA-DRB1*04:04 and HLA-B*08 cases, was the age at MG onset. DRB1*04:04 was associated with an earlier onset than B*08 (Table 4). Since an arbitrary cut of at age <12 years were used to define the groups, this support an association with true prepubertal onset. AChR ab positivity and thymus hyperplasia seemed more frequent among the HLA-B*08 cases; however, the sample size was too small to establish a true difference between the two groups of prepubertal onset.

The HLA-B*08 positive juvenile MG cases in our material showed the characteristics typical for adult EOMG; female preponderance, AChR ab positivity and thymus hyperplasia (**Table 3**). The similarity of postpubertal onset MG to adult EOMG has been addressed earlier [31], and our findings concerning clinical picture and HLA association could support postpubertal onset juvenile MG being a continuum of adult EOMG. Prepubertal onset MG on the other hand, could comprise a distinctive subset of the disorder as hypothesized by Matsuki et al [32], and further supported by the differences we observed in the HLA associations.

Several studies have described the clinical presentation of juvenile MG in Western populations [20, 31, 33–36]. A challenge when comparing these studies, is their heterogeneity due to discrepancy on upper age cut off for the designation juvenile MG. However, several studies have differentiated between prepubertal onset and postpubertal onset, and show differences in disease characteristics between the two groups. The prepubertal onset cases are associated with higher frequency of seronegativity, higher frequency of ocular MG but also some with severe disease although with good prognosis [1, 20, 31, 37]. Although subtle, the clinical differences together with the current HLA findings, could suggest that prepubertal onset MG constitute a distinctive subset of the disorder.

The main strength of this study is the comprehensive HLA genotyping done on a population based study cohort with extensive clinical mapping. However, the sample size is small due to the rarity of juvenile MG, and this limits the study. Further research including larger number of juvenile MG cases, especially those with prepubertal onset, is necessary to confirm the DRB1*04:04 association and to better describe the differences between the clinical subgroups.

In conclusion, this study provides novel information about HLA associations in European juvenile MG, where HLA-DRB1*04:04 is associated with prepubertal onset. In postpubertal onset juvenile MG, the HLA association is with HLA-B*08 like in EOMG.

## Supporting information

S1 Table Distribution of HLA alleles observed in the juvenile MG patients compared to controls. (DOCX)
