# Supplementary material for: Juvenile myasthenia gravis in Norway: HLA-DRB1*04:04 is positively associated with prepubertal onset
Source: PLoS One. 2017 Oct 16;12(10):e0186383. doi: 10.1371/journal.pone.0186383 (PMC5643110; doi:10.1371/journal.pone.0186383)
Supplement: S1 Table — (DOCX) [file pone.0186383.s001.docx]

**S1 Table**: Distribution of HLA alleles observed in the juvenile MG patients compared to controls

| **Locus** | **Allele** | **JMG**  **n=86** | **Controls**  **n=734** | **JMG**  **Freq** | **Control**  **Freq** |
| --- | --- | --- | --- | --- | --- |
| **HLA-A** | 01:01 | 27 | 119 | 0.314 | 0.1621 |
|  | 02:01 | 17 | 240 | 0.1977 | 0.327 |
|  | 03:01 | 13 | 116 | 0.1512 | 0.158 |
|  | 11:01 | 5 | 41 | 0.05814 | 0.05586 |
|  | 24:02 | 4 | 78 | 0.04651 | 0.1063 |
|  | 25:01/26:01/26:08 | 5 | 34 | 0.05814 | 0.04632 |
|  | 31:01/32:01 | 10 | 71 | 0.1163 | 0.09673 |
|  | 68:01 | 5 | 35 | 0.05814 | 0.04768 |
| **HLA-B** | 51:01 | 4 | 26 | 0.04651 | 0.03571 |
|  | 07:02 | 9 | 117 | 0.1047 | 0.1607 |
|  | 08:01 | 28 | 94 | 0.3256 | 0.1291 |
|  | 14:02 | 1 | 21 | 0.01163 | 0.02885 |
|  | 15:01/15:18 | 4 | 77 | 0.04651 | 0.1058 |
|  | 18:01 | 1 | 17 | 0.01163 | 0.02335 |
|  | 27:05 | 4 | 57 | 0.04651 | 0.0783 |
|  | 35:01 | 3 | 56 | 0.03488 | 0.07692 |
|  | 37:01 | 3 | 12 | 0.03488 | 0.01648 |
|  | 39:01 | 1 | 17 | 0.01163 | 0.02335 |
|  | 40:01/40:02 | 17 | 75 | 0.1977 | 0.103 |
|  | 44:02/44:03 | 8 | 110 | 0.09302 | 0.1511 |
|  | 47:01 | 1 | 1 | 0.01163 | 0.001374 |
|  | 55:01 | 1 | 4 | 0.01163 | 0.005495 |
|  | 57:01 | 1 | 21 | 0.01163 | 0.02885 |
| **HLA-C** | 01:02 | 3 | 28 | 0.03488 | 0.03966 |
|  | 02:02 | 4 | 45 | 0.04651 | 0.06374 |
|  | 03:03/03:04 | 20 | 130 | 0.2326 | 0.1841 |
|  | 04:01 | 3 | 73 | 0.03488 | 0.1034 |
|  | 05:01 | 3 | 56 | 0.03488 | 0.07932 |
|  | 06:02 | 5 | 39 | 0.05814 | 0.05524 |
|  | 07:01/07:02/07:04 | 42 | 246 | 0.4884 | 0.3484 |
|  | 08:02 | 1 | 37 | 0.01163 | 0.05241 |
|  | 12:03 | 2 | 21 | 0.02326 | 0.02975 |
|  | 15:02 | 2 | 13 | 0.02326 | 0.01841 |
|  | 16:01 | 1 | 13 | 0.01163 | 0.01841 |
| **HLA-DRB1** | 01:01 | 8 | 77 | 0.09302 | 0.1052 |
|  | 03:01 | 26 | 106 | 0.3023 | 0.1448 |
|  | 04:01 | 4 | 92 | 0.04651 | 0.1257 |
|  | 04:02 | 1 | 1 | 0.01163 | 0.001366 |
|  | 04:04 | 13 | 47 | 0.1512 | 0.06421 |
|  | 04:08 | 1 | 5 | 0.01163 | 0.006831 |
|  | 07:01 | 3 | 55 | 0.03488 | 0.07514 |
|  | 08:01 | 2 | 29 | 0.02326 | 0.03962 |
|  | 09:01 | 1 | 6 | 0.01163 | 0.008197 |
|  | 10:01 | 3 | 5 | 0.03488 | 0.006831 |
|  | 11:01 | 1 | 32 | 0.01163 | 0.04372 |
|  | 13:01 | 4 | 47 | 0.04651 | 0.06421 |
|  | 13:02 | 9 | 39 | 0.1047 | 0.05328 |
|  | 15:01 | 9 | 109 | 0.1047 | 0.1489 |
|  | 16:01 | 1 | 1 | 0.01163 | 0.001366 |
| **HLA-DQB1** | 02:01 | 26 | 137 | 0.3023 | 0.193 |
|  | 02:02 | 2 | 4 | 0.02326 | 0.005634 |
|  | 03:01 | 4 | 117 | 0.04651 | 0.1648 |
|  | 03:02 | 16 | 95 | 0.186 | 0.1338 |
|  | 03:03 | 2 | 26 | 0.02326 | 0.03662 |
|  | 04:02 | 2 | 32 | 0.02326 | 0.04507 |
|  | 05:01 | 11 | 95 | 0.1279 | 0.1338 |
|  | 05:02 | 1 | 1 | 0.01163 | 0.001408 |
|  | 06:02 | 9 | 103 | 0.1047 | 0.1451 |
|  | 06:03 | 4 | 47 | 0.04651 | 0.0662 |
|  | 06:04 | 7 | 35 | 0.0814 | 0.0493 |
|  | 06:09 | 2 | 1 | 0.02326 | 0.001408 |

JMG = juvenile myasthenia gravis cases

Freq = frequency
